# Determination of Young’s modulus of Sb_2_S_3_ nanowires by in situ resonance and bending methods

**DOI:** 10.3762/bjnano.7.25

**Published:** 2016-02-19

**Authors:** Liga Jasulaneca, Raimonds Meija, Alexander I Livshits, Juris Prikulis, Subhajit Biswas, Justin D Holmes, Donats Erts

**Affiliations:** 1 Institute of Chemical Physics, University of Latvia, Raina blvd 19, Riga, LV-1586, Latvia; 2 Materials Chemistry and Analysis Group, Department of Chemistry, University College Cork, Cork, Ireland; 3 CRANN & AMBER, Trinity College Dublin, Dublin 2, Ireland; 4 Tyndall National Institute, Lee Maltings, Cork, Ireland; 5 Department of Chemistry, University of Latvia, Raina blvd 19, Riga, LV-1586, Latvia

**Keywords:** antimony sulfide, in situ, mechanical properties, nanowires, Young’s modulus

## Abstract

In this study we address the mechanical properties of Sb_2_S_3_ nanowires and determine their Young’s modulus using in situ electric-field-induced mechanical resonance and static bending tests on individual Sb_2_S_3_ nanowires with cross-sectional areas ranging from 1.1·10^4^ nm^2^ to 7.8·10^4^ nm^2^. Mutually orthogonal resonances are observed and their origin explained by asymmetric cross section of nanowires. The results obtained from the two methods are consistent and show that nanowires exhibit Young’s moduli comparable to the value for macroscopic material. An increasing trend of measured values of Young’s modulus is observed for smaller thickness samples.

## Introduction

Antimony sulfide or stibnite is a highly anisotropic semiconductor material with potential applications in thermoelectric and optoelectronic [1–2] devices due to its high achievable thermoelectric power and photosensitivity [3], its large absorption coefficient [4–5] and direct band gap in the visible and near infrared range (1.78–2.5 eV) [6–8]. Owing to these properties, Sb_2_S_3_ has also been considered as an attractive material for microwave frequency [9], optical recording [10] and photovoltaic [2,11] applications. It has also been studied as a photonic bandgap material in the visible region of the electromagnetic spectrum [12].

Synthesis and characterization of various Sb_2_S_3_ nanostructures including dendrites [13], nanorods [14], whiskers [15], nanowires (NWs) [16–17] and nanotubes [18] have been reported. Particular emphasis has been placed on the investigation of structural and optical properties of Sb_2_S_3_ thin films [19–20]. Recently, piezoelectricity and ferroelectricity has been demonstrated in individual single-crystalline Sb_2_S_3_ NWs embedded in anodic aluminum oxide (AAO) templates [17]. However, published measurements of the mechanical properties of Sb_2_S_3_ nanostructures are very scarce. To our knowledge there are no reports about experimental investigation of mechanical properties of individual Sb_2_S_3_ cantilevered nanostructures and their Young’s modulus in particular.

Understanding the elastic behavior of nanostructures is not only important for the development of potential applications, but also allows for an overall estimation of the NW structure obtained using different synthesis methods. Various approaches have been used to study the mechanical behavior of NWs including AFM 3-point bending test [21–22] and nanoindentation [23]. In situ techniques stand out among other methods for mechanical characterization due to their capability of real-time monitoring of the elastic response of the NWs. Bending tests with a use of external force sensor [24], tensile deformation [25–26] as well as mechanical [27–28] and thermal [29] resonance have all been successfully used to determine the mechanical properties. These techniques allow for the measurement of elastic parameters such as hardness and Young’s modulus, as well as for the investigation of the plasticity of individual NWs [30].

Three different trends have been outlined in existing literature when measuring the basic mechanical parameters of NWs. Firstly, recent studies report increasing Young’s modulus with diminishing size of the nanostructure [28,31]. Secondly, the opposite, namely an increasing size of the nanostructure leading to a decrease of Young’s modulus for thinner NWs [32] has also been observed. Finally, it has been found that some materials exhibit no dependence of the Young’s modulus on size [21–22].

It is therefore of great interest to find out to what extent the observed discrepancies are intrinsic to the examined NWs and how to account for the differences in the measurement methods. Substantial uncertainties in mechanical tests may arise from variations in boundary conditions and several methods have been proposed to resolve this issue [33–34]. Moreover, inhomogeneous cross section, amorphous outer layer and curvature of the NW may result in additional errors. Bending methods using the AFM may suffer from slippage of the AFM tip over NW and the effects arising from the induced force in axial direction in case of double clamped NWs [21–22]. For in situ tensile testing precise alignment of the NW is a crucial factor and the method involves laborious preparation of the specimen. Electrically induced mechanical resonance is a facile method that offers relatively fast characterization of the NW but caution has to be taken when determining the true resonance frequency as other factors such as inhomogeneous mass distribution may be responsible for unwanted resonant excitation.

In the present study we report measurements of Young’s modulus of free standing Sb_2_S_3_ NWs. Our experiments were comprised of two different methods of mechanical investigation of the NWs inside the chamber of a scanning electron microscope (SEM). One method was the electric-field-induced mechanical resonance, while the other involved static bending of Sb_2_S_3_ NWs with atomic force microscope (AFM) tip inside SEM. The choice of the methods for this study allowed for subsequent characterization of the same NW without changing other experimental conditions (e.g., clamping) than alignment of the external force. The obtained mean value was then compared to that of bulk material [35].

## Experimental

The examined Sb_2_S_3_ NWs were synthesized inside cylindrical pores of anodic aluminum oxide template (AAO) by a solvent-less technique. The as-synthesized NWs were single-crystalline and showed orientation along *c*-axis as revealed by high resolution transmission electron micrograph images and selected area electron diffraction pattern analysis [17]. A detailed description of NW synthesis can be found in [17]. Sb_2_S_3_ NW powder was obtained by mechanically removing outgrown NWs from the surface of the AAO template. Alternatively NWs from dissolved AAO templates were also used for mechanical testing. In this case filled AAO templates with NW diameters ranging from 80 to 200 nm were polished, dissolved in 9% H_3_PO_4_, washed and dried.

The as-prepared Sb_2_S_3_ NW powder was used to glue NWs to an Au probe with conductive epoxy CW2400 to obtain free-standing single-clamped NW specimens. A SmarAct 13D nanomanipulation system with an additional vacuum-compatible micro stepper-motor (Faulhaber ADM0620) was staged into a field emission scanning electron microscope (SEM Hitachi S4800) for resonance and static bending experiments. The micro stepper-motor was used for rotation of the specimen for exact determination of NW cross-sectional dimensions and for precise positioning of the NW-countering tip system. For mechanical resonance measurements, an AC signal generator (Agilent N9310A) and a DC voltage source (Keithley 6430) were connected to probes mounted on nanomanipulation system. A frequency tunable AC voltage provided by a signal generator was applied across the NW and the countering Au tip. At excitations near the natural resonance frequency of the NW, mechanical oscillation of the NW can be easily monitored directly in SEM images. For static bending experiments, soft silicon nitride AFM cantilevers with spring constants of 0.002–0.02 N/m (Olympus BL-RC-150VB) were used. The spring constant of the cantilever was calibrated in AFM (Asylum Research MFP-3D BIO) using the thermal noise method [36].

## Results and Discussion

**Figure 1:**
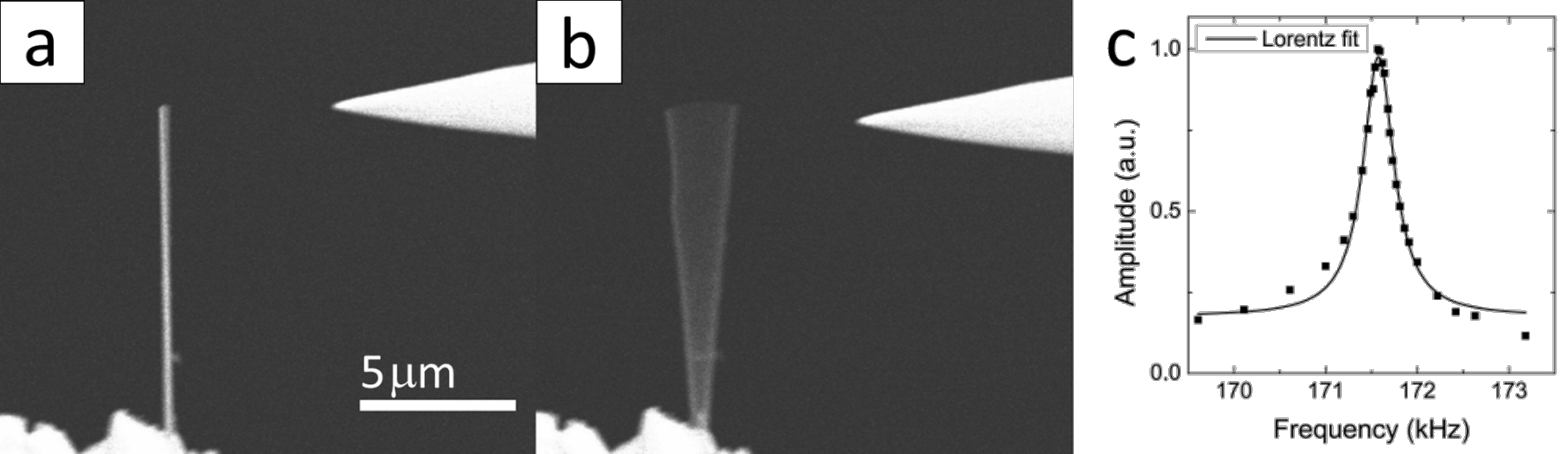
In situ resonance excitation of Sb_2_S_3_ NW. SEM images of NW with dimensions: length *L* = 10 μm and radius *r* = 67 nm recorded when the applied electric field frequency is a) far from the natural resonance frequency of the NW and b) at the resonance frequency. c) Amplitude-frequency curve for Sb_2_S_3_ NW with ω_0_/2π = 171.57 kHz and *Q* = 418.

Figure 1a,b shows SEM images of Sb_2_S_3_ nanowires with a length of 10 μm and a radius of 67 nm with applied AC voltage at two different frequencies, where one is far from the resonance and second matches resonant excitation. The force acting on the NW is proportional to the electric field squared [37], thus both cos(ω*t*) and cos(2ω*t*) components can be observed. Depending on which term dominates the resonance, the driving frequency, ω, either equals the fundamental resonance frequency of the NW or corresponds to one half of the NW resonance frequency. Therefore oscillations at one half and at double the resonance frequency were examined for each NW to ensure that the true resonance frequency has been found.

Quality factors, *Q*, were determined for each NW by measuring oscillation amplitude versus frequency. In Figure 1c, an amplitude–frequency curve is plotted for a typical single-clamped Sb_2_S_3_ NW with *Q* = 418. Damping ratios in resonance experiments for all NWs were in the range of 0.001–0.003, hence their contribution to the observed resonance frequencies and consequently calculated Young’s modulus values was negligible.

**Figure 2:**
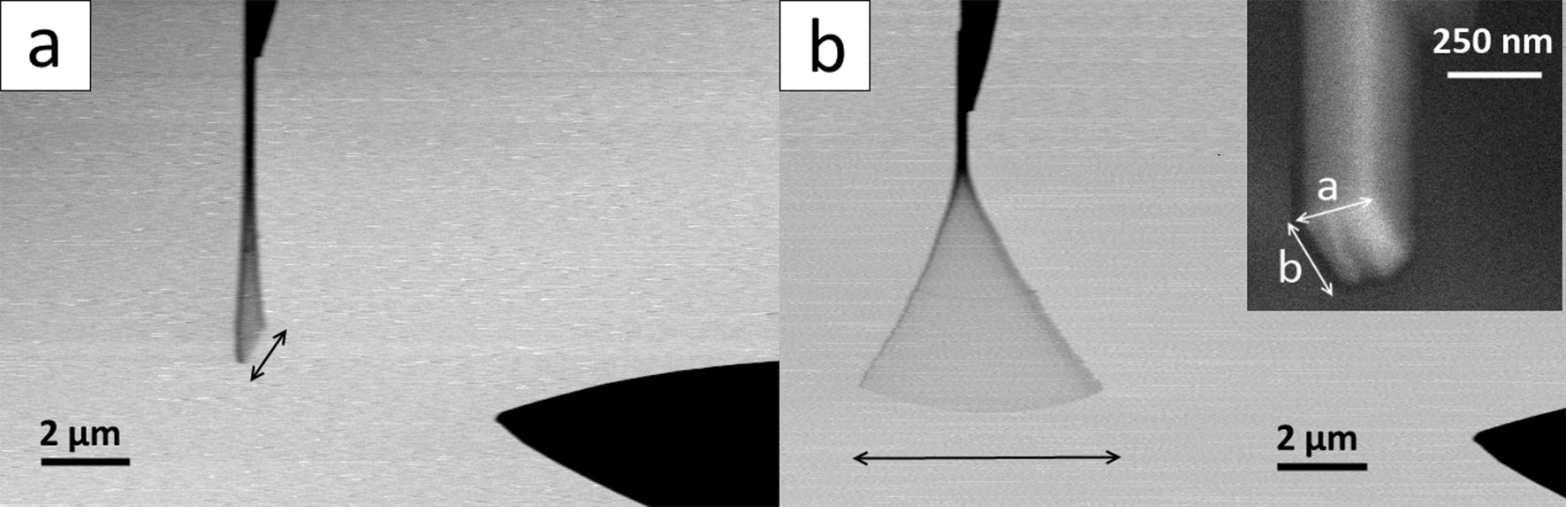
SEM images of resonantly excited Sb_2_S_3_ NW with rectangular cross-section, showing two mutually orthogonal directions of oscillation: a) nearly perpendicular with the direction of the driving electric field and b) parallel to the direction of the electric field. Large NW deflections are used for illustrative purposes. Inset shows rectangular cross-section of NW.

The resonance frequency was determined for 20 Sb_2_S_3_ NWs with lengths ranging from 6.6 to 30 μm and average thickness from 120 to 305 nm. However, SEM observations revealed that the investigated NWs had either circular or rectangular cross-sections. Resonance in mutually perpendicular directions was observed for NWs with rectangular cross-sections (Figure 2). It was experimentally confirmed that the ratios of the resonance frequencies ω_1_/ω_2_ were consistent with the corresponding ratios *a*/*b* of the NW side lengths (Table 1). Based on the measured fundamental resonance frequency, ω*_n_*, and dimensions of the NW, the Young’s modulus, *E*, was calculated using the expression for the resonance frequency 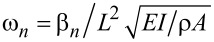 according to Euler–Bernoulli dynamic beam theory [38]. Here *L* is the length of the NW, *A* the cross-sectional area, ρ is the density of Sb_2_S_3_, β_0_ = 1.875 for the first resonant mode and the area moments of inertia are given as *I* = π/4(*d*/2)^4^ and *I* = *ab*^3^/12 for NWs with circular and rectangular cross-section, respectively. Here, *d* denotes diameter of NWs with circular cross-section and *a* and *b* are side lengths for NWs with rectangular cross-section. Small vibrational amplitudes (less than 10% of *L*) were used during resonance to be consistent with the Euler–Bernoulli bending theory assumptions. SEM characterization confirmed that all examined NWs had constant cross-sectional area along their length, which allows for the application of Euler–Bernoulli equations.

**Table 1:** Ratios of resonance frequencies ω_1_/ω_2_ show consistency with corresponding ratios *a/b* of NW side lengths.

*a*/*b*	ω_1_/ω_2_

0.80 ± 0.05	0.81 ± 0.03
0.83 ± 0.10	0.76 ± 0.06
0.84 ± 0.10	0.87 ± 0.07

**Figure 3:**
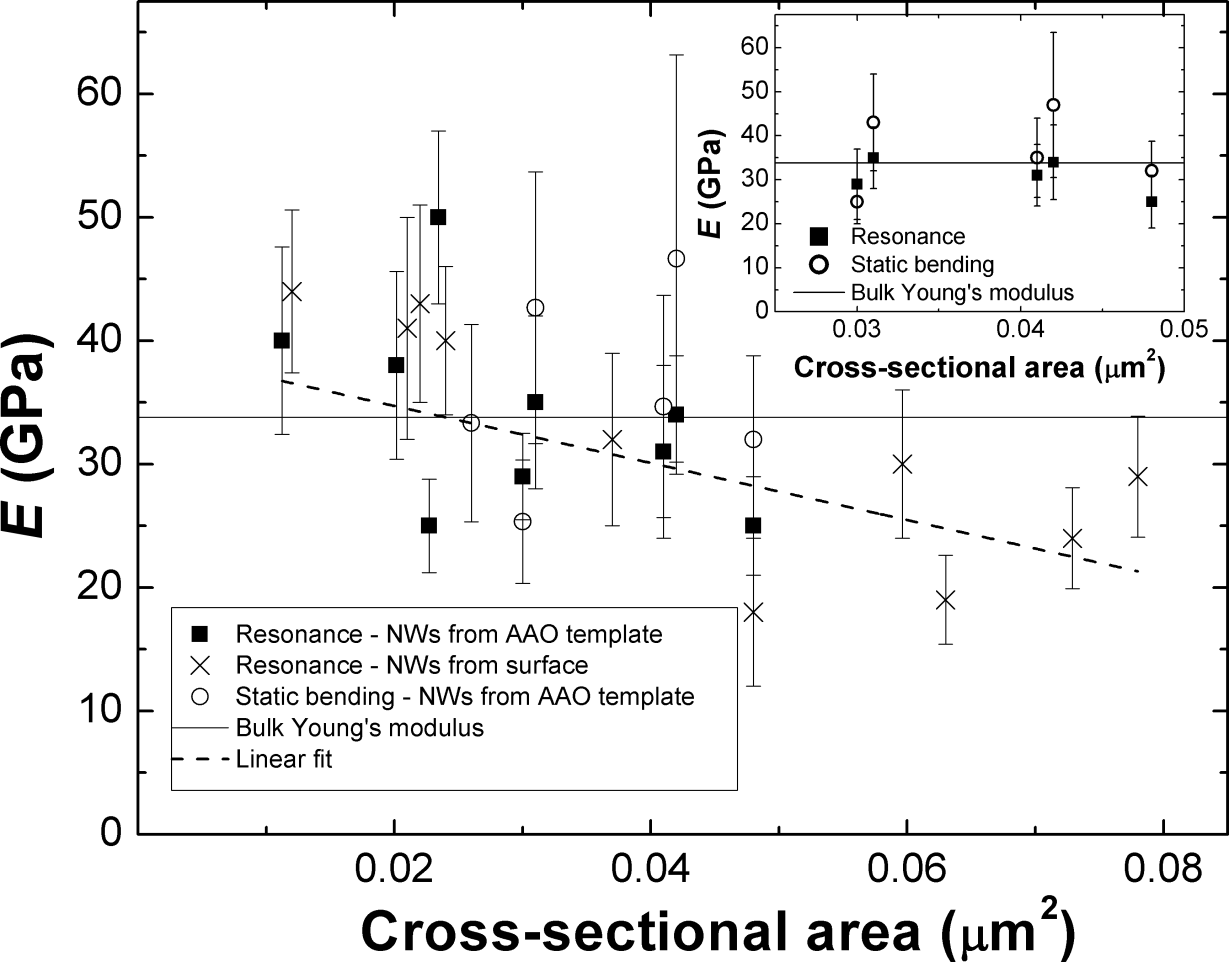
Young’s modulus of Sb_2_S_3_ NWs as a function of their size. Data points represent the measured Young’s modulus values determined from mechanical resonance and static bending experiments as a function of NW cross-sectional area. The continuous line at 33.8 GPa is used as an estimate for the Young’s modulus of crystalline Sb_2_S_3_ in *c*-axis direction. The dashed line is a linear fit added to experimentally obtained data to highlight the size effect. Inset: Experimentally determined Young’s modulus values of five Sb_2_S_3_ NWs using both static bending (circles) and mechanical resonance (squares) techniques. Both methods give similar results within experimental accuracy.

The dependence of the Young’s modulus of Sb_2_S_3_ NWs on their size is revealed in Figure 3 by plotting the measured Young’s modulus values as a function of the cross-sectional area of the NWs. The experimentally obtained Young’s modulus values are in the range of 18–50 GPa with Young’s modulus of NWs with larger cross-sectional area (more than 0.06 µm^2^) lying below the value of 33.8 GPa, which corresponds to the Young’s modulus of crystalline Sb_2_S_3_ in the direction of the *c*-axis, calculated using the speed of sound along the *c*-axis (2.71·10^5^ cm·s^−1^ [35]). As the cross-sectional area gets smaller, the values of Young’s modulus tend to increase. A first principles study has been carried out on Sb_2_S_3_ compound [39] obtaining a value for the speed of sound along the longitudinal direction of approximately 5.46·10^5^ cm·s^−1^, which corresponds to a Young’s modulus value of 121.50 GPa. The theoretically calculated value of Young’s modulus for bulk crystal is approximately four times larger than that obtained in the present study for NWs and that for bulk material [35]. Figure 3 also reveals that the Young’s modulus of NWs grown inside nanopores of an AAO matrix and those outgrown on the surface are very similar within experimental accuracy.

**Figure 4:**
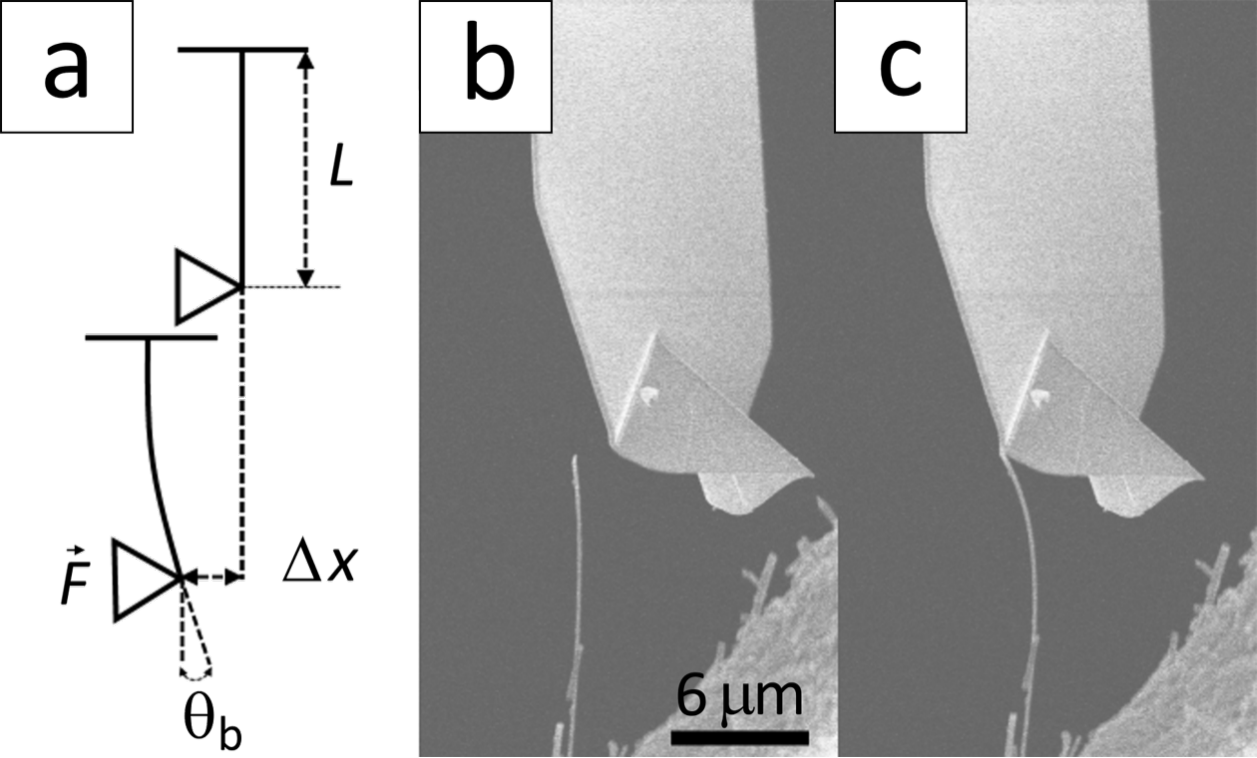
a) Schematic of the static bending of a single Sb_2_S_3_ NW. SEM images of the NW b) in a relaxed state and c) under bending load.

To assess the accuracy of the obtained results and exclude possible errors associated with the applied resonance method, alternative mechanical testing was done by determining the Young’s modulus using static bending for some of the NWs that were examined by resonance method. During in situ bending test NW was pushed against the tip of the cantilever. The applied load direction was adjusted perpendicular to the vertical axis of the NW. Two SEM images were recorded for each nanowire during the bending, namely one under bending load and the other one in a relaxed state. By overlapping the two images both the displacement of the AFM tip, *∆x*, and the angle of rotation, θ_b_, at the loading point of the NW were measured, which allowed for the calculation of the NW bending force, *F*. The schematics of the bending experiment and a typical SEM image recorded during the manipulation are shown in Figure 4a and Figure 4b–c, respectively. The NW was oriented perpendicular to the direction of the electron beam as it is essential to ensure that the real displacement is measured, instead of a projection at an unknown angle.

Knowing the angle of rotation, θ_b_, the applied bending load, *F*, the vertical position of the applied load and dimensions of the NW, the Young’s modulus, *E*, was calculated using Euler–Bernoulli’s static bending equation for a cantilevered beam *E* = *Fy*^2^/2θ_b_*I* [40]. Here, *y* is the vertical position of the applied load and *I* is the area moment of inertia. The applied bending load was calculated using the measured cantilever displacement, *∆x*, and the cantilever spring constant, *k*, as F = *k*·Δ*x*. For static bending the load was applied at different vertical positions along the vertical axis of the NW. The measurements showed that the examined NWs exhibited uniform elastic properties along their length.

The inset in Figure 3 shows a comparison between results obtained by resonance and static bending methods for five of the examined NWs. It can be seen that both methods give similar results. The discrepancies between the obtained results could be assigned to errors in measuring displacement and length of the NWs. By solving *E*_resonance_(*L*) = *E*_bending_(*L*) for *L*, the calculated value was compared with experimentally obtained results, giving a mean relative error of 31%. Measurement errors due to cantilever spring constant calibration were taken into account.

Figure 3 suggests that a size effect exists for the investigated NWs over the examined cross sectional area range, with the apparent stiffness increasing for NWs with smaller cross sectional area. A linear fit added to the data points marks the tendency with a negative slope of Δ*E*/Δ*A* ≈ 230 GPa/μm². This can be explained by a nanoscale surface effect that arises from surface atoms being in a different environment than the bulk. An increasing surface-to-volume ratio may lead to the observed stiffening trend that has been described using a number of mechanisms such as surface reconstruction [28], surface bond saturation [41] and bulk nonlinear elasticity [42]. Additionally, the variation of the surface-to-volume ratio among the NWs with different shapes could contribute to the experimentally obtained scatter. It is also important to note that different loading methods may give rise to different elastic response of NWs. In this study we are dealing with similar mechanical loading where one side of the NW is being under compression while the other is under tension, which agrees well with the consistent results between the methods.

## Conclusion

We have experimentally obtained Young’s modulus of individual Sb_2_S_3_ NWs combining two different techniques, namely mechanical resonance and static bending. The results show that the investigated NWs have Young’s moduli close to that of bulk Sb_2_S_3_, which depend on the size over the examined range of the NW cross-sectional area. The scatter of the obtained values could be attributed to errors in measured geometrical parameters and different cross sectional geometries of the NWs, imperfectly defined boundary conditions and sliding at the NW–tip contact point in bending experiments.
